# Late-Onset HSV-2 Encephalitis in a Kidney Transplant Recipient: A Rare Case Report

**DOI:** 10.3390/life15020152

**Published:** 2025-01-22

**Authors:** Danijela Zjačić Puljiz, Ivana Vrkić, Ivo Jeličić, Dijana Borić Škaro, Ivana Kristina Delić Jukić, Lučana Vicelić Čutura, Mirela Pavičić Ivelja

**Affiliations:** 1 Clinic of Internal Medicine, Department of Nephrology, Dialysis and Hypertension, University Hospital Split, 21000 Split, Croatia; 2 School of Medicine, University of Split, 21000 Split, Croatia; 3 Department of Infectious Disease, University Hospital Split, 21000 Split, Croatia; 4 Clinic of Internal Medicine, Department of Hematology, University Hospital Split, 21000 Split, Croatia

**Keywords:** kidney transplant, HSV-2, encephalitis, immunosuppression

## Abstract

Infections are an important cause of morbidity and mortality in renal transplant recipients. Among the viral pathogens encountered in this population, herpes simplex virus (HSV), a member of the Alphaherpesvirinae subfamily, has an important place. HSV type 2 infections in this immunosuppressed population are primarily due to viral reactivation. While HSV-2 frequently presents as genital herpes or remains asymptomatic, in rare cases, it can lead to severe neurological manifestations, such as encephalitis, particularly in the early post-transplant period with a reported mortality rate of up to 40%. We present the case of a 49-year-old male who, three years after kidney transplantation, developed acute neurological symptoms, including aphasia and disorientation. Polymerase chain reaction (PCR) analysis of cerebrospinal fluid (CSF) identified HSV-2 as the causative pathogen, enabling a swift and accurate diagnosis. The patient was promptly treated with intravenous acyclovir, adjusted for renal function, resulting in complete neurological recovery and subsequent negative follow-up CSF PCR results. This case emphasizes the vital role of PCR diagnostics as the gold standard for confirming viral encephalitis, particularly in immunosuppressed patients, where atypical presentations can complicate diagnosis. It also highlights the importance of considering HSV-2 encephalitis in the differential diagnosis even beyond the immediate post-transplant period. Early recognition and management, facilitated by the multidisciplinary approach, are critical for improving outcomes in this vulnerable patient population.

## 1. Introduction

Infections are an important cause of morbidity and mortality in renal transplant recipients, with more than 80% of patients experiencing at least one infectious complication within the first year following transplantation [1,2].

In the early posttransplant period, infections are primarily associated with perioperative complications and nosocomial pathogens, often mitigated by prophylactic measures. The intermediate phase, which spans from one to six months post-transplantation, represents a period of heightened vulnerability due to the peak effects of immunosuppressive therapy. Opportunistic infections, including fungal, viral, bacterial, and parasitic pathogens, are particularly common in this phase, but also geographic and institutional factors, such as local epidemiology, immunosuppressive protocols, and antimicrobial prophylaxis strategies, play an important role in the incidence and type of infections encountered [1]. Also, the intensification of immunosuppression, often necessitated by graft rejection or conditions such as leukopenia or neutropenia, can prolong or reintroduce this high-risk period. The late phase, defined as six to twelve months or more post-transplantation, is marked by stabilization of immunosuppressive regimens, usually at reduced levels. Despite this stabilization, transplant recipients remain susceptible to community-acquired bacterial and viral infections. Viral infections continue to pose challenges during this phase, functioning not only as direct pathogens but also as cofactors that exacerbate other conditions or increase susceptibility to opportunistic infections [1].

Among the viral pathogens encountered in this specific population, *Cytomegalovirus* (CMV), *Epstein–Barr virus* (EBV), and *Herpes simplex virus type-1* and *type-2* (HSV-1 and HSV-2) are the most prevalent [1]. Herpes simplex virus (HSV), a member of the Alphaherpesvirinae subfamily, has a seroprevalence of approximately 60% in the general population, whereas the incidence of infection among renal transplant recipients is around 53%, placing these individuals at significantly increased risk [3]. HSV-1 infection, which is in most cases presented as classic orolabial herpes virus, is required from early age to adulthood with a prevalence in the United States of 80% by the age of 60 [4]. In the general population, HSV-2 has a seroprevalence that rapidly rises at the age of sexual debut, with the prevalence of 26.3% by the age of 49 [4]. Among transplant patients, the prevalence reflects these trends, as they are typically exposed to the viruses prior to transplantation [3].

In renal transplant recipients, HSV type 2 infections most commonly result from viral reactivation, though donor-to-recipient transmission, while rare, can also occur. Cytotoxic T lymphocytes (CTLs), a subset of T-cells, play a crucial role in controlling HSV reactivation from latency. CTLs recognize HSV-infected cells through the presentation of viral antigens via MHC class I molecules, triggering targeted destruction of these infected cells. Immunosuppressive therapies inhibit T-cell signaling pathways, leading to reduced CTL activity and impaired ability to contain viral replication. This immunosuppression creates an environment in which HSV can escape immune surveillance and reactivate [5].

The incidence of HSV reactivation in relation to specific immunosuppressive regimens has not been comprehensively evaluated.

However, historical data suggest that certain agents, such as the anti-CD3 monoclonal antibody muromonab (OKT3) and mycophenolate mofetil, may be associated with an increased risk of HSV reactivation following transplantation [6]. To date, no studies have directly compared different induction regimens—such as T cell-depleting agents (e.g., rabbit-antithymocyte globulin or alemtuzumab) versus non-depleting agents (e.g., basiliximab or daclizumab)—or maintenance immunosuppressive regimens regarding HSV reactivation rates. However, some evidence suggests that the use of mTOR inhibitors (e.g., rapamycin) in combination with reduced calcineurin inhibitor exposure may lead to a decrease in herpes virus infections [6].

Herpes simplex virus (HSV) commonly presents as orolabial, genital, or perianal disease. Lesions are often characterized by vesicles or ulcers, which may spread locally. In some cases, HSV can lead to visceral or disseminated infections, including widespread mucocutaneous involvement, esophagitis, hepatitis, or pneumonitis. The most frequent symptoms of disseminated disease include fever and leukopenia. Pneumonitis is observed in recipients of various organ transplants, with the highest prevalence in heart–lung transplant patients. Rarely, visceral infections may occur without any visible skin or mucosal lesions, posing a diagnostic challenge [3].

Clinically, HSV-2 infection commonly presents as genital herpes or remains asymptomatic, with mild, often overlooked symptoms. Imunosuppressed patients, compared to imunocompetent patients have more frequent and severe clinical manifestations and may have slower response to therapy [3].

In rare instances, HSV-2 may present with severe neurological manifestations such as encephalitis followed by seizures. Neuropathological symptoms, particularly due to the virus’s predilection for the limbic system, often manifest as behavioral changes and speech disturbances, including aphasia [7].

While most patients with herpes simplex virus (HSV) infection present with characteristic orolabial or genital lesions, the clinical manifestations in immunocompromised individuals can be atypical, necessitating laboratory confirmation for accurate diagnosis. HSV demonstrates robust growth in tissue culture, enabling identification of most isolates within a few days. However, the timing of sample collection is critical, particularly for mucocutaneous lesions. For instance, samples from genital lesions older than five days have a diagnostic yield of less than 35% [7].

Direct fluorescent antibody (DFA) offers rapid diagnostic results. Polymerase chain reaction (PCR) assays, which are up to four times more sensitive than tissue culture for detecting mucocutaneous HSV, have supplanted viral culture as the preferred diagnostic method. Nevertheless, culture and DFA remain viable options for mucocutaneous lesions [7].

For diagnosing HSV encephalitis, PCR testing of cerebrospinal fluid is the gold standard, exhibiting a sensitivity of 98%. HSV DNA has also been detected in the blood of immunocompetent patients with primary ulcerative infections and in those experiencing significant reactivation. However, the clinical relevance of detecting HSV DNA in blood outside of cases with disseminated disease remains unclear [8].

Magnetic resonance imaging (MRI) is a highly sensitive test (over 90%) for detecting herpes simplex encephalitis. Lesions appear hypodense on T1 and hyperintense on T2 and FLAIR, often affecting the temporal lobes, having progressed along the limbic system to the inferior frontal lobes and insular cortex. High-resolution FLAIR can reveal thalamic changes not visible on DWI, while the basal ganglia are usually unaffected. Immunocompromised patients may show atypical patterns outside the frontotemporal regions and often have widespread brain involvement, including the brainstem and cerebellum, but in one-third of patients, there are no pathological changes on MRI [8].

Serological testing is seldom useful for diagnosing acute infections; most patients are HSV-seropositive, and IgM positivity may indicate viral reactivation rather than new infection. However, IgG-based serological testing is valuable in pre-transplant assessments to stratify post-transplant risk.

The gold standard for diagnosis of encephalitis caused by HSV is polymerase chain reaction (PCR) analysis of cerebrospinal fluid (CSF) [8].

In this case report, we describe a rare instance of HSV2 encephalitis in a kidney transplant recipient, highlighting the clinical presentation and treatment outcomes.

To our knowledge, this is the first reported case of HSV-2 encephalitis occurring in the late post-transplant period following kidney transplantation.

## 2. Case Report

A 49-year-old male with a history of renal transplantation from a deceased donor in April 2020 presented to the emergency department in October 2023 with sudden-onset aphasia and disorientation.

The etiology of his underlying renal disease was unknown. His medical history was notable for arterial hypertension and depression, both of which were well-controlled with standard therapies. The patient had been maintained on a standard immunosuppressive regimen, including basiliximab for induction and a combination of tacrolimus, mycophenolate mofetil, and corticosteroids for maintenance therapy. His graft function was very good and remained stable with serum creatinine levels consistently around 200 µmol/L, and no episodes of acute rejection had been documented. Prior to transplantation, the patient was seropositive for HSV, and post-transplant antiviral prophylaxis with valganciclovir was administered for a duration of six months, alongside standard prophylactic therapy.

Upon presentation in our emergency department, the patient had a Glasgow Coma Scale (GCS) score of 7. He was afebrile, conscious, disoriented, eupneic, and hemodynamically stable, with an oxygen saturation of 98% on room air and a blood pressure of 136/88 mmHg. There were no changes on the skin and genital area, urine output was normal, and abdominal ultrasonography showed no signs of urine retention. Neurological examination revealed no focal deficits beyond aphasia and disorientation.

Initial laboratory investigations showed a white blood cell count of 6.2 × 10^9^/L, normocytic anemia, a platelet count of 226 × 10^9^/L, and stable renal function with urea at 19.7 mmol/L and creatinine at 231 µmol/L. Lactate dehydrogenase (LDH) was slightly elevated at 270 U/L, while liver function tests were normal. Inflammatory markers were low, with a C-reactive protein (CRP) of 1.5 mg/L; procalcitonin was also in the normal range. The patient’s tacrolimus level was 6.5 µg/L, and his estimated glomerular filtration rate (eGFR) was 30 mL/min/1.73 m^2^.

The analysis of cerebrospinal fluid showed elevated erythrocyte levels (106 × 10^6^/L) without pleocytosis. Initial imaging, multislice computed tomography (MSCT), revealed that brain parenchyma exhibits appropriate densities both supra- and infratentorially, with midline-positioned ventricular structures of normal size and shape, without visible displacement. No focal lesions were identified. There were no signs of acute ischemia or hemorrhage.

**Figure 1:**
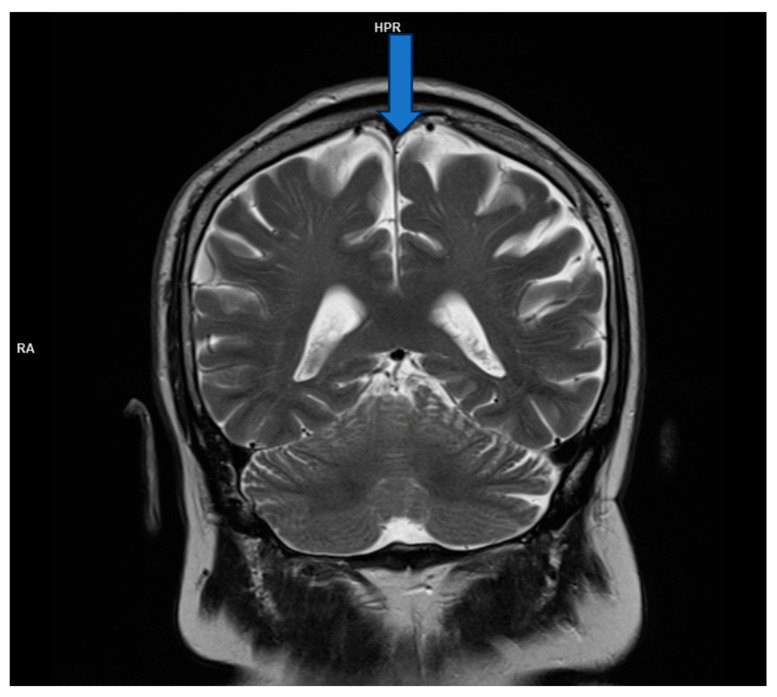
MRI; T2 coronal section- cortical atrophic changes.

**Figure 2:**
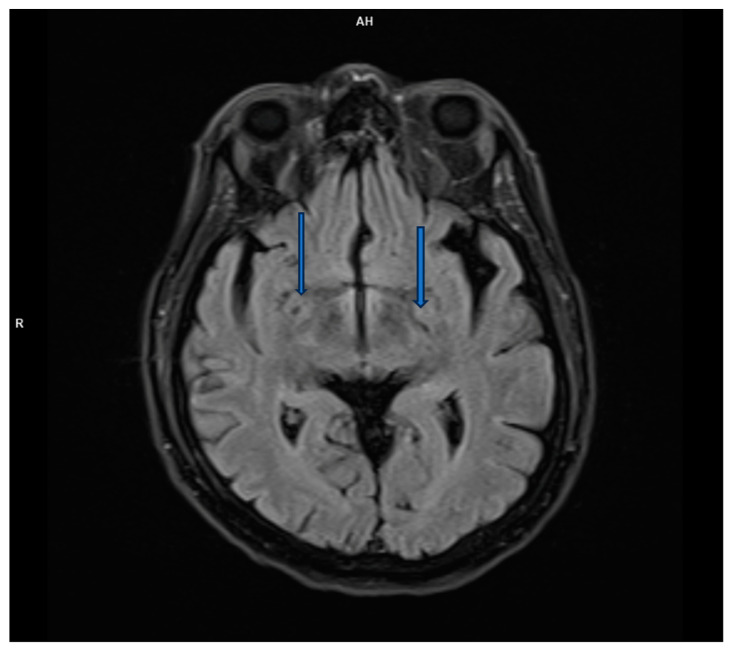
FLAIR transverse MRI scan demonstrates prominent perivascular spaces.

**Figure 3:**
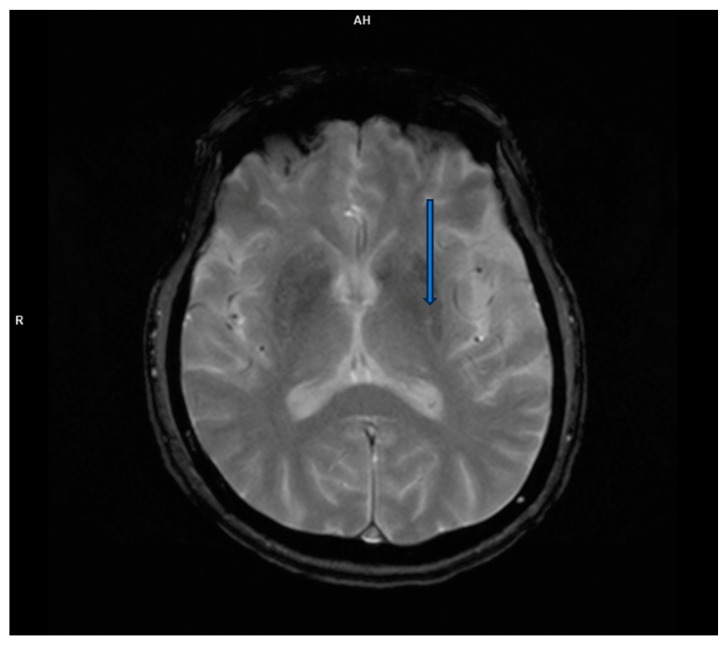
T2-weighted transverse MRI scan reveals enlarged perivascular spaces.

However, brain magnetic resonance imaging (MRI) revealed diffuse cortical atrophy with widened cerebrospinal fluid spaces and prominent perivascular spaces bilaterally in the subinsular region (Figure 1, Figure 2 and Figure 3).

Cerebrospinal fluid (CSF) analysis via polymerase chain reaction (PCR) confirmed the presence of herpes simplex virus type 2 (HSV-2), establishing the diagnosis of HSV-2 encephalitis.

The patient was immediately started on intravenous acyclovir, with the dose adjusted for renal function.

The hospital course was complicated by the development of fever accompanied by an increase in CRP levels (213.6 mg/L). Radiographically, pneumonia was confirmed, and empirical therapy with piperacillin-tazobactam was initiated. A bronchoscopy was performed, during which no signs of secretion were observed endobronchially. Tests for *BCG*, *M. tuberculosis*, and PCR multiplex were negative. A bronchoalveolar lavage was performed, and *Pneumocystis jirovecii* was not detected. The culture of the bronchial aspirate was positive for *E. coli*. Blood, CSF, and urine cultures were negative. Tests for *Cytomegalovirus* (CMV) and *Mycobacteria* also returned negative. On the 7th day of hospitalization, in the cerebrospinal fluid, there was no pleocytosis, but there was a slight elevation in lactate and red blood cells.

During hospitalization, the patient developed leukopenia (white blood cell count of 0.9 × 10^9^/L) with associated lymphopenia. Filgrastim was administered to stimulate leukocyte production. Bone marrow cytology revealed relative lymphopenia, consistent with a viral infection. In response, mycophenolate mofetil was discontinued, steroid therapy was increased to 15 mg daily, and tacrolimus was maintained within the therapeutic range (approximately 4 µg/L).

The patient demonstrated clinical improvement over the course of treatment, with normalization of inflammatory markers and full neurological recovery.

After 14 days of therapy with acyclovir, follow-up CSF PCR testing was negative for HSV-2. The patient was discharged in stable condition, without any residual neurological deficits.

## 3. Discussion

Herpesviridae is a leading cause of viral encephalitis after kidney transplantation [9]. HSV type 1 (HSV-1). HSV type 2 (HSV-2), although more commonly associated with genital herpes, accounts for 2–10% of encephalitis cases and carries a high mortality rate, reaching up to 40% in untreated cases [3]. The occurrence of HSV-2 encephalitis in immunocompromised patients, such as solid organ transplant recipients, is rare but life-threatening. These epidemiological findings align with the results of a French study by Tamzali et al., which revealed that viral encephalitis accounts for 24.5% of cases among kidney transplant recipients, with 10.6% attributed to HSV-1 or HSV-2 [9]. In solid organ transplant recipients, HSV infection typically results from the reactivation of a latent virus, with clinical manifestations often limited to mucocutaneous lesions. However, disseminated or neurologically invasive HSV infections, such as encephalitis, are particularly severe in this population [8]. It is crucial to determine the serostatus prior to transplantation, as HSV-seropositive recipients face a significant risk of clinical reactivation post-transplant if antiviral prophylaxis is not administered, even in the absence of prior clinical HSV disease. Among HSV-seropositive adult transplant patients who do not receive antiviral prophylaxis, the incidence of clinically evident HSV disease ranges between 35% and 68% [6]. The risk is further elevated due to immunosuppressive regimens, which may impair the immune response to viral reactivation. Although the incidence of HSV encephalitis in transplant recipients is lower compared to the general population, those who do develop encephalitis face significantly higher morbidity and mortality [10,11]. The clinical hallmark of HSV encephalitis is the involvement of the limbic system, leading to behavioral disturbances, seizures, and speech disorders such as aphasia. Presentation also includes headache, and alteration of sensorium with signs attributable to affected CNS parenchyma or cranial nerves [12,13].

In the described case, the patient presented with a sudden onset of aphasia and disorientation, which are hallmark neurological manifestations of HSV-2 encephalitis. Prompt recognition of these symptoms, alongside the utilization of polymerase chain reaction (PCR) testing of cerebrospinal fluid (CSF), was crucial in establishing a definitive diagnosis. PCR analysis of CSF is widely regarded as the gold standard for diagnosing HSV encephalitis due to its high sensitivity and specificity, enabling the confirmation of the viral etiology in a timely and reliable manner. Numerous studies have focused on developing models and criteria to optimize the use of PCR testing in resource-limited settings, and they have proposed and validated criteria for deferring HSV PCR testing in immunocompetent patients with normal cerebrospinal fluid (CSF) white blood cell counts (≤5 cells/mm^3^), and protein levels (≤50 mg/dL). However, these criteria apply exclusively to immunocompetent patients; for transplant recipients, PCR remains the gold standard [14]. The early initiation of antiviral therapy significantly impacts the clinical course of HSV encephalitis, a potentially life-threatening condition. Treatment involves the administration of high-dose intravenous acyclovir, which is effective in controlling viral replication and mitigating the risk of severe neurological damage. In cases where HSV demonstrates resistance to acyclovir, alternative therapies such as foscarnet or cidofovir are recommended [15]. However, both medications are linked to considerable renal toxicity, necessitating careful monitoring to manage the associated risks of these alternative therapies [6]. Failure to initiate treatment promptly can lead to catastrophic outcomes, including permanent cognitive deficits, severe disability, or death [16]. Post-treatment prophylaxis with oral acyclovir is advised to reduce the risk of HSV recurrence. However, there are no established guidelines specifying the duration of follow-up after an episode of HSV encephalitis. Instead, patients typically continue with routine post-transplant monitoring, conducted at intervals of 1 to 3 months [17].

In our case, the patient responded well to acyclovir, administered in a dose adjusted for renal function, in alignment with current treatment guidelines.

Despite the success of antiviral therapy, the patient developed leukopenia during the treatment, requiring adjustments in immunosuppressive therapy and the administration of filgrastim. The leukopenia may have been caused by a combination of factors, including chronic therapy, acute infection, and the use of acyclovir. This highlights the delicate balance required in managing immunosuppressed patients to control both infection and potential complications from immunosuppression.

Corticosteroid therapy remains controversial in the treatment of HSV encephalitis [18]. While some studies, such as the GACHE trial, explored the combination of acyclovir and corticosteroids, they did not conclusively demonstrate superiority in clinical outcomes [19]. In the case presented, corticosteroids were increased due to concerns about inflammation, which may have contributed to the patient’s favorable recovery. Numerous studies suggest supportive treatment with IVIG, especially in cases of hypogammaglobulinemia, which is common in transplant patients [20].

Wagner et al. conducted a systematic review covering 377 studies and 39 case reports (17 involving transplant patients), concluding that IVIG therapy is safe, but the decision to use it remains individual [20]. In our case, the decision to forgo intravenous immunoglobulin (IVIG) therapy, despite its reported benefits in transplant patients with hypogammaglobulinemia, was based on the patient’s robust response to conventional antiviral therapy. The development of new antiviral agents, such as amenamevir and pritelivir, opens promising avenues for future research. Both are helicase-primase inhibitors that have shown superior efficacy compared to traditional antivirals like acyclovir and ganciclovir in treating VZV and HSV infections, with a favorable safety profile. Pritelivir has demonstrated effectiveness in hematopoietic stem cell transplant (HSCT) recipients with acyclovir-resistant HSV-2 genital herpes, while amenamevir has shown synergistic effects with acyclovir and ganciclovir in vitro, enhancing its potential for managing severe viral infections [15,21,22]. Notably, oral amenamevir has been approved in Japan for the treatment of herpes zoster (HZ) and the prevention of post-herpetic neuralgia. However, to date, neither of these antivirals has been studied in solid organ transplant recipients (SOTRs), but they undoubtedly hold promise for the future [15].

To our knowledge, this is a unique case of HSV-2 encephalitis in the late post-transplant period following kidney transplantation. The literature review reveals no reports of transplant patients with HSV-2 encephalitis.

Uehara et al. described seven cases of HSV infection, all occurring 3–38 months post-transplantation, with none presenting as encephalitis [23]. Ramirez et al. reported a case of HSV-1 encephalitis in a kidney transplant patient [24]. Basse et al. described three cases of disseminated HSV infection in SOT in the early post-transplant period, but none presented as encephalitis [25].

This case is notable as it demonstrates the occurrence of HSV-2 encephalitis more than three years post-transplant, emphasizing the necessity for ongoing vigilance regarding opportunistic infections well beyond the early high-risk period. It highlights the critical importance of maintaining a high index of suspicion for HSV-2 encephalitis in patients presenting with acute neurological decline. Early recognition is pivotal, as the disease can have devastating neurological consequences if not promptly diagnosed and treated. The use of rapid diagnostic tools, such as polymerase chain reaction (PCR) testing of cerebrospinal fluid, is essential for confirming the diagnosis, while immediate initiation of antiviral therapy plays a key role in improving patient outcomes.

This case serves as a reminder for clinicians to remain alert to atypical or subtle presentations of HSV encephalitis, which may deviate from classical symptoms, ensuring timely recognition and management to maximize the potential for favorable outcomes.

## 4. Conclusions

HSV-2 encephalitis is an extremely rare but serious infective complication in kidney transplant recipients. While most cases occur early in the post-transplant period, this report highlights the possibility of late-onset disease, emphasizing the importance of maintaining a high index of suspicion for opportunistic infections at any time post-transplant.

The multidisciplinary approach to the immunosuppressed patient enables early diagnosis and prompt antiviral therapy, which are essential for improving patient outcomes. Considering the growing number of immunosuppressed, kidney-transplanted patients, further research is needed to better understand the epidemiology and optimal management of HSV-2 encephalitis in this vulnerable population, especially those beyond the early post-transplant period.

## Data Availability

The data presented in this paper are available on request from the corresponding author.
